# The role of stimulus-driven versus goal-directed processes in fight and flight tendencies measured with motor evoked potentials induced by Transcranial Magnetic Stimulation

**DOI:** 10.1371/journal.pone.0217266

**Published:** 2019-05-20

**Authors:** Agnes Moors, Chiara Fini, Tom Everaert, Lara Bardi, Evelien Bossuyt, Peter Kuppens, Marcel Brass

**Affiliations:** 1 Research Group of Quantitative Psychology and Individual Differences, KU Leuven, Leuven, Belgium; 2 Centre for Social and Cultural Psychology, KU Leuven, Leuven, Belgium; 3 Department of Experimental Clinical and Health Psychology, Ghent University, Ghent, Belgium; 4 Department of Dynamic and Clinical Psychology, State University of Roma “La Sapienza”, Rome, Italy; 5 Department of Experimental Psychology, Ghent University, Ghent, Belgium; University of Bologna, ITALY

## Abstract

This study examines two contrasting explanations for early tendencies to fight and flee. According to a stimulus-driven explanation, goal-incompatible stimuli that are easy/difficult to control lead to the tendency to fight/flee. According to a goal-directed explanation, on the other hand, the tendency to fight/flee occurs when the expected utility of fighting/fleeing is the highest. Participants did a computer task in which they were confronted with goal-incompatible stimuli that were (a) easy to control and fighting had the highest expected utility, (b) easy to control and fleeing had the highest expected utility, and (c) difficult to control and fleeing and fighting had zero expected utility. After participants were trained to use one hand to fight and another hand to flee, they either had to choose a response or merely observe the stimuli. During the observation trials, single-pulse Transcranial Magnetic Stimulation (TMS) was applied to the primary motor cortex 450 ms post-stimulus onset and motor-evoked potentials (MEPs) were measured from the hand muscles. Results showed that participants chose to fight/flee when the expected utility of fighting/fleeing was the highest, and that they responded late when the expected utility of both responses was low. They also showed larger MEPs for the right/left hand when the expected utility of fighting/fleeing was the highest. This result can be interpreted as support for the goal-directed account, but only if it is assumed that we were unable to override the presumed natural mapping between hand (right/left) and response (fight/flight).

## Introduction

**Fig 1:**
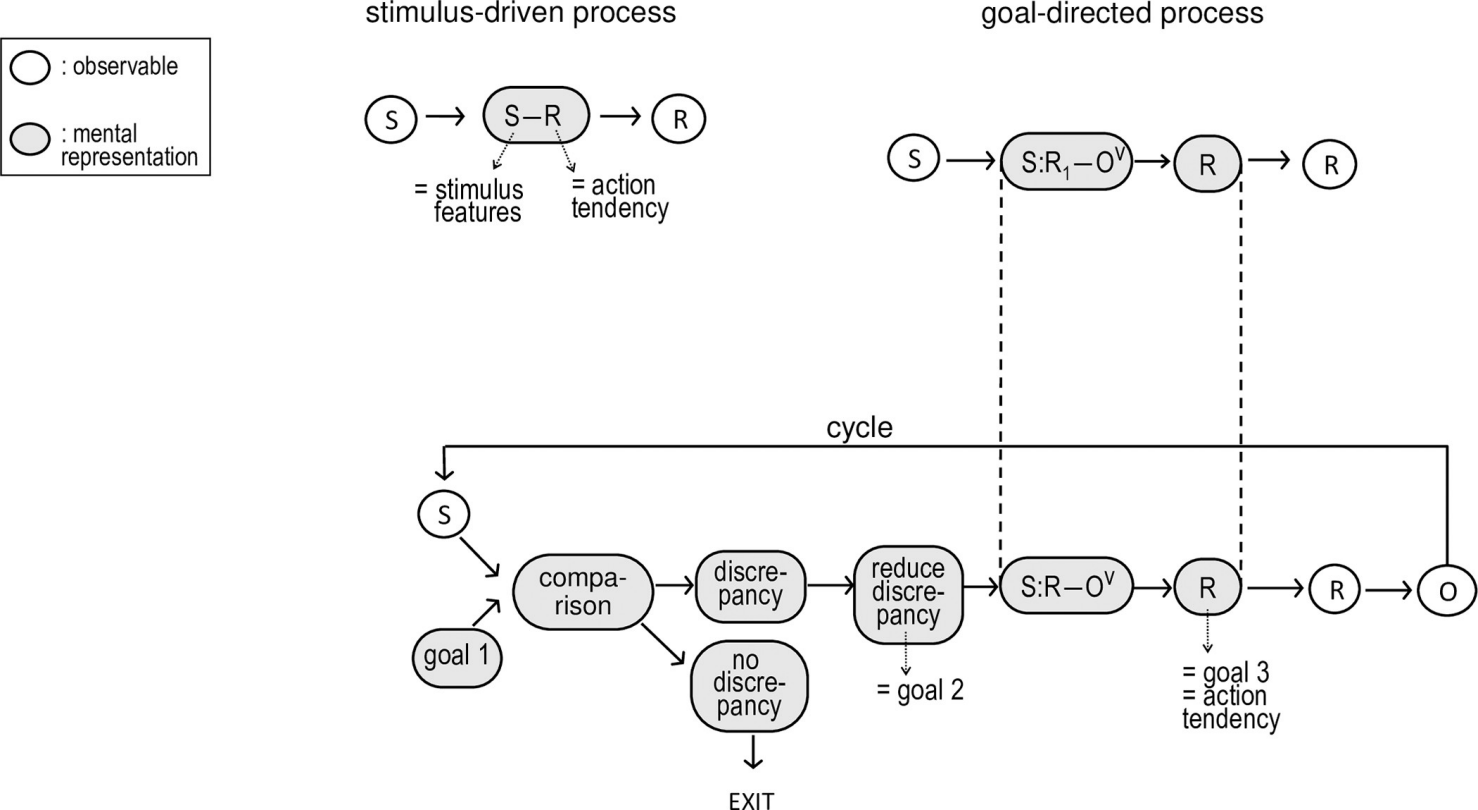
**Visual representation of the stimulus-driven** (top left) **and goal-directed mechanism** (top right)**, and of the embedding of the goal-directed mechanism in an action control cycle** (bottom).

Many (aversive) emotional encounters are characterized by behaviors that qualify as fighting (i.e., aggressive or offensive behavior) or fleeing (i.e., avoidant, defensive, or safety seeking behavior), or at least by the tendencies to engage in these behaviors. A barking dog makes us want to flee. A demeaning offense makes us want to fight. Many existing accounts of emotional behavior follow a dual process logic. They explain this behavior by the interplay of stimulus-driven and goal-directed processes, two types of processes that have been defined in terms of the content of their mediating representations [1–3] (see Fig 1).

In a stimulus-driven process, a stimulus activates an association between the representation of stimulus features (S) and the representation of a response (R). The latter representation can also be called an action tendency. Stimulus features can range from more perceptual (e.g., loudness of noise) to more abstract ones, including ones that refer to the stimulus-person interaction (e.g., goal in/compatibility of the stimulus). A common stimulus-driven hypothesis is that stimuli that are evaluated as negative or goal-incompatible and difficult/easy to control lead to the tendency to flee/fight [4]. This hypothesis stems from appraisal theories of emotion, which hold that evaluation of a stimulus as goal-incompatible and difficult/easy to control by the person leads to anger/fear [4–9], and that anger/fear is typically characterized by the tendency to fight/flee [10–12]. As Ellsworth and Scherer ([4], p. 580) put it: “In the case of an obstructive stimulus brought about by a conspecific aggressor or predator, the comparison between the organism’s estimate of its own power and the agent’s perceived power is likely to decide between anger and fear and thus between fight and flight.” This aligns with Keltner, Gruenfeld, and Anderson’s ([13]; see also [14]) proposal that low power activates the behavioral inhibition system (BIS), responsible for the tendency to avoid, whereas high power activates the behavioral approach system (BAS), responsible for the tendency to aggress.

A goal-directed process, on the other hand, assesses the expected utility of one or more action options in the current situation. This process is mediated by representations of the *expectancy* that certain actions will lead to certain outcomes (R-O) and the *values* of these outcomes (O^v^) given a certain stimulus (S). The action option with the highest expected utility (value x expectancy) activates its corresponding action tendency (R), and this action tendency can be manifested in overt behavior [15]. The goal-directed process does not occur in a vacuum but is best embedded in a cycle [2–3], in which the detection of a discrepancy between a stimulus (actual state) and a first goal (i.e., representation of a valued outcome) gives the impetus to a second goal to reduce the discrepancy. If a discrepancy is detected, the organism strives to reduce the discrepancy (i.e., a *second* goal), either by acting (i.e., assimilation), by choosing a different first goal (i.e., accomodation), or by reinterpreting the stimulus (i.e., immunization) [16]. The utility of acting and of specific action options determines whether an action will be chosen and which one. The outcome of the action feeds back to the comparator where it constitutes the stimulus input to the next cycle, and the cycle is repeated until the discrepancy is resolved. To illustrate, if a person encounters an enemy who poses a discrepancy with her goal for safety, this activates a second goal to reduce this discrepancy. If the person estimates that fighting has a higher expectancy than fleeing to regain her safety, she will activate the tendency to fight rather than to flee.

A sharp observer may note that the stimulus-driven and the goal-directed accounts of fight/flight behavior present some overlap. Indeed, the evaluation of goal in/compatibility in the stimulus-driven account corresponds to the evaluation of a/no discrepancy in the goal-directed account. In addition, the evaluation of control in the stimulus-driven account corresponds to the overall expectancy of the action options in a person’s repertoire. Despite this overlap, however, both accounts are not redundant. The appraisal of a stimulus as easy/difficult to control refers to whether the overall expectancy of the action options is low/high but it does not specify which specific action option has the highest expectancy. This means that there are cases in which both processes predict the same action tendencies and cases in which they predict different action tendencies. In typical cases in which control is low (e.g., a conflict in which the person is weaker than the opponent), fleeing may be the optimal action, but there may be situations in which this is not true. For instance, control can be so low that even fleeing is impossible. Likewise, fighting may be the optimal action in a typical high-control situation, but again, this may not always be true. For instance, a person may have high control in the sense that she can flee but not fight.

Evidence for the stimulus-driven hypothesis that goal-incompatible stimuli that are easy/difficult to control lead to the tendency to fight/flee comes from self-report studies. For instance, Frijda, Kuipers, and ter Schure [17] reported a correlation between modifiability of a stimulus and the tendency to go against someone. Evidence also comes from experimental studies in which high-power individuals engaged more in aggressive behavior than low-power individuals [13, 18–20]. There is also evidence against this hypothesis, however. Some studies show that low control leads to more rather than less aggression [21–22]. Other studies show that high social power leads to a higher degree of activity, regardless of whether this activity was antisocial or prosocial [23].

These mixed results have led researchers to turn to two strategies: The first strategy is to refine the factor of control, either by marking the distinction with other related factors (e.g., power, dominance, authority) or by splitting it into subtypes (stable vs. situational control, control by oneself vs. anyone), and to argue that different factors or subtypes lead to different action tendencies. The second strategy is to invoke moderators. One type of moderators are other abstract stimulus features (i.e., appraisal factors) such as expectedness, agency, type of goal, or legitimacy [24]. Another type of moderators are stable person factors such as communal vs. exchange relationship orientation [25] and high vs. low self-esteem [18]. A final type of moderators are other processes, such as the presumed goal-directed processes involved in emotion regulation [26] and in the planning of concrete behavior [27].

By invoking goal-directed regulation (and planning) processes, researchers commit to a dual process model with a default-interventionist architecture. Such a model assumes that the stimulus-driven process is the default determinant of behavior and goal-directed processes only intervene under special conditions. This assumption is rooted in the idea that stimulus-driven processes are automatic, which means that they can occur under all conditions including poor conditions (e.g., little time and attentional resources, and the lack of an intention to engage in the process), whereas goal-directed ones are nonautomatic, which means that they can only occur under ample conditions (e.g., abundant time and attentional resources, and the intention to engage in the process) [28]. Applied to the fight/flight case, the initial tendency to fight/flee is determined by a stimulus appraised as goal incompatible and easy/difficult to control (i.e., a stimulus-driven process), but this tendency can later be regulated by a goal-directed process that compares the expected utilities of fighting and fleeing. If an initial tendency to fight or flee is not possible (e.g., fleeing is not possible because one is trapped) or not desirable because it conflicts with some goal (e.g., fighting conflicts with the goal to keep a relationship intact), the goal-directed process can intervene to suppress this tendency.

Recently, Moors [2–3] proposed an alternative dual process model with a parallel-competitive architecture. The model assumes that not only stimulus-driven but also goal-directed processes can operate automatically, so that both processes often operate in parallel and compete with each other. The model, moreover, assumes that the goal-directed process often wins the competition and gets to determine the lion share of the action tendencies, including the initial ones. Applied to the fight/flight case, the initial tendency to fight/flee should already be based on a weighting of the expected utilities of different action options (for a similar view, see [29]).

Taken together, the two types of dual process models have different assumptions regarding the processes responsible for the initial tendency to fight/flee. The default-interventionist model predicts that this tendency will be determined by a stimulus-driven process, such as the process that links goal-incompatible stimuli that are easy/difficult to control to the tendency to fight/flee. The parallel-competitive model predicts that this tendency will be determined by a goal-directed process that weights the expected utilities of fighting and fleeing.

To pit the stimulus-driven and goal-directed accounts of initial fight/flight tendencies against each other, we manipulated the overall ease/difficulty to control a stimulus, partially independently from the expected utilities of the specific actions of fighting and fleeing, and we measured the participants’ initial action tendencies.

To manipulate the appraisal of overall *ease/difficulty to control* a stimulus, we presented three goal-incompatible stimuli that were contrasted with regard to their objective controllability: One stimulus was impossible (i.e., extremely difficult) to control whereas the other two were easy to control. To manipulate the *expected utilities* of fighting and fleeing, we manipulated the expectancy that these actions would lead to a valued outcome by manipulating the objective likelihoods that they would lead to this outcome. For the stimulus that could not be controlled, the expectancies of both fighting and fleeing were zero. For the stimuli that were easy to control, one stimulus had a high expectancy for fighting (and a zero expectancy for fleeing) whereas the other had a high expectancy for fleeing (and a zero expectancy for fighting).

To measure the initial action tendencies elicited by these stimuli, the methods of self-report and behavioral choice tasks are not suitable because the effects obtained with these methods may reflect the influence of goal-directed processes in a later stage. Stimulus-response compatibility tasks relying on reaction times are generally considered to be more suitable for measuring early action tendencies (e.g., approach-avoidance tendencies), but even this method is not free from regulatory influences [30]. To circumvent the shortcomings of behavioral methods, we developed a variant of the neurophysiological method that measures Motor Evoked Potentials (MEPs) induced by Transcranial Magnetic Stimulation (TMS) of the primary motor cortex (M1). We did also measure behavioral choices, partly as a manipulation check (see below).

TMS over M1 induces an electrical current in the cortex, which is passed on to the cortico-spinal tract to the peripheral motor neurons, where it produces a MEP (a peak in EMG activity 20 to 50 ms after the pulse). The amplitude of this MEP is considered as an index of the excitability of the cortico-spinal tract [31], which allows for the detection of motor preparation or action tendencies even in the absence of overt behavior.

Previous research with single-pulse motor TMS already provides information about the factors that contribute to the activation of emotional action tendencies. Some studies examined the influence of appraisals of valence [32–36], goal in/compatibility [37–39], and agency [40] on action tendencies, in line with a stimulus-driven account. Other studies examined the influence of values and expectancies of action options [41–43] on action tendencies, in line with a goal-directed account. Apart from the fact that these studies did not directly pit stimulus-driven and goal-directed accounts of their findings against each other, most of them have only measured a general increase or decrease in hand MEPs, suggesting a general increased or decreased readiness to act. For instance, Borgomaneri, Gazzola, and Avenanti [32] found that (right-hemisphere) TMS during the presentation of both positive and negative body postures led to a decrease in MEP. Avenanti, Minio-Paluello, Sforza, and Aglioti [37] found that seeing pain stimuli being administered to another person’s hand led to a decrease in MEPs in the same hand but an increase in MEPs in the opposite hand. Some researchers have interpreted a general decrease and increase in MEPs as indicative of specific action tendencies. For instance, Borgomaneri et al. [32] and Avenanti [35] linked a decrease in MEPs to the tendency to freeze or orient and Avenanti et al. [37] linked an increase in MEPs to the tendency to avoid. It could be argued, however, that a mere decrease in MEPs is also compatible with any other “passive” tendency (e.g., the tendency to give in) and a mere increase in MEPs with any other “active” tendency (e.g., the tendency to fight). In other studies, specific action tendencies have not been inferred from a general increase or decrease in MEPs but instead from MEPs in specific muscles. For instance, Gough, Campione, and Buccino [44] linked MEPs in the first dorsal interrosseus (FDI) of the index finger to approach because it is involved in grasping, and MEPs in the extensor communis digitorum (EC) of the forearm to avoidance because it is involved in releasing one’s grasp. It could be argued, however, that grasping is not uniquely linked to approach (e.g., when you grasp a safety figure to avoid a threat) and that releasing grasp is not uniqely linked to avoidance (e.g., when you release your grasp from a safety figure to approach a threat).

The present study goes beyond previous TMS research in two ways. First, it aims to directly pit a stimulus-driven account against the goal-directed account. Second, it aims to measure specific action tendencies instead of a general increase or decrease in action readiness and in a way that does not assume a fixed relation between specific muscle activity and high-level actions. While in previous research, specific high-level action tendencies have been *inferred*, either from a general increase or decrease in MEPs or from MEPs in specific muscles, we chose instead to *install* the meaning of specific action tendencies during a training phase. Specifically, before administering TMS, we trained participants to use the index finger of one hand to fight and that of the other hand to flee. Assuming that this training would establish connections between low-level motor movements (finger presses) and high-level meaningful actions (fight and flight), the comparison of the MEPs at the FDIs of both hands should allow us to conclude which action tendency was activated most strongly in a given condition.

The current experiment was a multiple-trial computer game in which participants encountered four avatars: one giver, who gave money to the participants and hence was compatible with the participants’ goal to win points, and three thieves, who tried to steal the participants’ money and hence were incompatible with the participants’ goal to win points. After participants were trained to press one key with the left hand to produce one action (e.g., fight) and another key with the right hand to produce the other action (e.g., flee), they learned that one thief could never be defeated (difficult to control, low expectancy of fighting and fleeing), another thief could only be defeated by fighting (easy to control, high expectancy of fighting), and still another thief could only be defeated by fleeing (easy to control, high expectancy of fleeing). In the test phase, each of a series of trials started with the visual presentation of one of the four avatars (yielding information about in/compatibility, ease/difficulty to control, and expectancies of fighting and fleeing). Participants were instructed to choose a response, but only after they were presented with a response cue (1000 ms post-stimulus onset). These response trials were intermixed with observation trials, in which a response cue did not appear and participants merely had to observe the stimuli. During observation trials, a TMS pulse was delivered over the left/right primary motor cortex (M1) at 450 ms post-stimulus onset, and the ensuing MEP from the right/left index fingers were registered. The response trials were added to keep the mapping between hand and response strong throughout the experiment, and as a manipulation check.

The stimulus-driven hypothesis predicts that the two thieves that are easy to control will elicit the tendency to fight (larger MEPs in the fight finger than in the flee finger) whereas the thief that is impossible to control will elicit the tendency to flee (larger MEPs in flee finger than in the fight finger). The goal-directed hypothesis predicts that the thief for which fighting has the highest expectancy will elicit the tendency to fight, the thief for which fleeing has the highest expectancy will elicit the tendency to flee, and the thief for which fighting and fleeing have zero expectancy will elicit the tendency to be passive. The stimulus-driven and goal-directed accounts thus make contrasting predictions (a) for the thief that is easy to control by fleeing (stimulus-driven account the tendency to fight; goal-directed account the tendency to flee) and (b) for the thief that is difficult to control (stimulus-driven account the tendency to flee; goal-directed account the tendency to be passive). Both accounts make the same prediction for the thief that is easy to control by fighting (the tendency to fight).

## Method

### Participants

According to a power analysis using G*Power [45], the required sample size to detect an effect of medium size of *f* = .25 under standard criteria (*α* = 0.05 two-tailed, *β* = 0.95, rcorr. = 0.5, nonsphericity correction = 1) is 28. We tested 30 healthy students recruited at Ghent University (15 females). Their age ranged from 18 to 30 years (*M* = 22.23 ± 2.53). All had normal or corrected-to-normal vision and all but two were right handed. All participants were checked for TMS exclusion criteria [46] and gave their written informed consent before participation. The study was in accordance with the Declaration of Helsinki and approved by the local ethics committee of Ghent University. Each participant received a compensation of 30 euro at the end of the experiment.

### Materials

Participants were seated at a distance of 60 cm from a 17-inch computer monitor with a keyboard vertically located on the table in front of them. The visual stimuli consisted of black silhouettes combined with 4 faces selected from the Radboud face database, developed by Langner et al. [47]: Face 7 for the giver, and Faces 24, 30, and 33 for the thieves. This selection was based on a prior rating study in which we took a subset of 20 Caucasian faces, categorized as neutral by at least 80% of the sample of Langner et al. [47], and in which we asked participants to rate the physical strength (N = 51) and the criminal look (N = 57) of these faces on a scale ranging from 1 (physically weak/not at all criminal) to 7 (physically strong/very criminal). The faces for the thieves were not significantly different in valence (*M*_*24*_ = 3.24; *M*_*30*_ = 3.33; *M*_*33*_ = 3.21) according to the Langner et al. [47] study, and our own rating study indicated that they were not significantly different with regard to physical strength (*M*_*24*_ = 4.33, SD = 1.14; *M*_*30*_ = 4.55, SD = 0.92; *M*_*33*_ = 4.53, SD = 1.16), *t(50)*s < 1.30, *p* = .20, and criminal look (*M*_*24*_ = 3.49, SD = 1.47; *M*_*30*_ = 3.35, SD = 1.22; *M*_*33*_ = 3.12, SD = 1.30), *t(56)*s < 1.57, *p* = .12. Participants wore headphones through which auditory response cues were presented. The experiment was programmed with Affect 4.0 software [48].

### Procedure

The experiment had the format of a computer game in which participants tried to earn money as a street artist. During the entire game, a hat with money lying on the street was presented in the lower half of the screen. At the beginning of the experiment, the hat contained 30 euro. The experiment consisted of (a) a S-O practice phase, in which participants learned stimulus-outcome associations, (b) a hand-R practice phase, in which they learned the mapping between hands and responses, (c) a S:R-O practice phase, in which they learned response-outcome contingencies (i.e., expectancies) given a certain stimulus, and (d) a test phase, in which the learned S:R-O relations still held and in which overt response choices were measured during response trials and covert action tendencies with TMS/MEP during observation trials. Each of these phases is described in detail below.

#### S-O practice phase

In this practice phase, participants encountered four different avatars. They learned that during a later phase (the test phase), one avatar (the giver) would give them money (2 euro) and hence would signal a positive outcome, whereas three other avatars (the thieves) would steal money (1 euro) from them and hence would signal a negative outcome. In this phase, no money was lost or won yet. The task of the participants was merely to observe the visual scenes and to remember which avatar was the giver and which avatars were the thieves. The phase comprised one block of 20 trials, 5 trials for each avatar. Each trial started with the presentation of an avatar (a black standing silhouette with a picture of a face in the head part) in front of the hat. The giver crouched towards the hat after 2000 ms and moved his hand to the hat while a money-dropping sound was played (this animation took 200 ms). He then remained crouched for another 800 ms before disappearing and the message “+2” together with an upward-pointing green arrow was presented for 1000 ms. The trials with thieves followed the same sequence of events as the trials with the giver, except that after a thief had crouched, a money-grabbing sound was played and the message “-1” together with a red arrow pointing downwards was displayed. The ITI was set to 1000 ms on average (with a 500 to 1500 ms range). To make sure that participants learned the S-O relations, they were asked at the end of this phase to identify the three thieves in a line-up together with the giver by clicking with the mouse on the thieves’ faces. When participants made a mistake, the experimenter told them and asked them to try again until they gave the correct response.

#### Hand-R practice phase

In this practice phase, participants learned the mapping between a hand or key (left vs. right) and a response (fight vs. flee). A faceless silhouette appeared on the screen, and participants were instructed via an auditory response cue to either fight (“vecht” in Dutch) or flee (“vlucht” in Dutch). The vertically located keyboard had only four keys of which two were horizontally aligned closest to the monitor (the close keys) and two were horizontally aligned farthest away from the monitor (the far keys). Participants kept their left and right index fingers on two (peripheral) resting keys when they were not responding, and they pushed the (central) response keys next to the resting keys to respond. Half of the participants received the instruction to use their right hand to fight and their left hand to flee. They fought by moving their right index finger from the close-right key to the adjacent close-central key labeled with the word “vecht” and fled by moving their left index finger from the far-right key to the adjacent far-central key labeled with the word “vlucht”. The other half received the opposite instruction to use their left hand to fight and their right hand to flee. They fled by moving their right index finger from the far-right key to the adjacent far-central key labeled with the word “vlucht” and fought by moving their left index finger from the close-right key to the adjacent close-central key labeled with the word “vecht”. In both hand-R mappings, participants fought with the hand closest to the screen and fled with the hand farthest away from the screen. In this way, we sought to increase the match with natural fight and flight responses given that fighting/fleeing is an approach/avoidance response and is more natural to perform with the hand close to/far from the target. Correct fight responses were followed by an animation (of 700 ms) in which a fist hit the thief and a punching sound was played. Correct flee responses were followed by an animation (of 500 ms) in which a hand grabbed the hat and a running-with-money sound was played. The response deadline was 2000 ms. If participants answered too late, the message “TOO LATE!” appeared on screen for 1000 ms and the thief stole the money. If participants answered incorrectly (i.e., they fought/fled after they heard a flee/fight cue), the message “ERROR!!!” appeared on screen for 1000 ms and the thief stole the money. The ITI was the same as in the previous phase. This practice phase comprised three trials with a fight instruction and three trials with a flee instruction, randomly intermixed.

#### S:R-O practice phase

In this final practice phase, participants learned S:R-O links both through verbal instruction and experience. They were instructed that a first thief could be defeated by fighting (he was presented as fast but weak, so he would not steal money if the participant would fight him). This thief was easy to control and fighting had the highest expected utility (i.e., easy-fight condition). A second thief could be defeated by fleeing (he was presented as slow but strong, so he would not steal money if the participant would flee from him). This thief was easy to control and fleeing had the highest expected utility (i.e., easy-flee condition). A third thief could never be defeated (he was presented as fast and strong, so he would steal money irrespective of the participant’s response). This thief was difficult to control and fleeing and fighting had zero expected utility (i.e., difficult condition). Participants were instructed to try to remember which responses (if any) were most effective to defeat the thieves.

This practice phase comprised 30 trials with 10 trials for each condition (easy-fight, easy-flee, and difficult). Each of the thieves was followed an equal number of times by a fight cue and a flee cue after which participants had to fight or flee. The fight response was followed by a punching fist and sound and the flee response by a grabbing of the hat and sound (like in the key-R practice phase). After this, if participants had correctly followed the instructions, the thief remained static and their money was not stolen. If they had made a mistake, however, the thief crouched and stole their money (conveyed by a downward pointing red arrow and “-1”). The thief/giver crouched 1000 ms after being presented, and the feedback appeared 1000 ms after that. The ITI was again the same as in the previous phase.

#### Test phase

**Fig 2:**
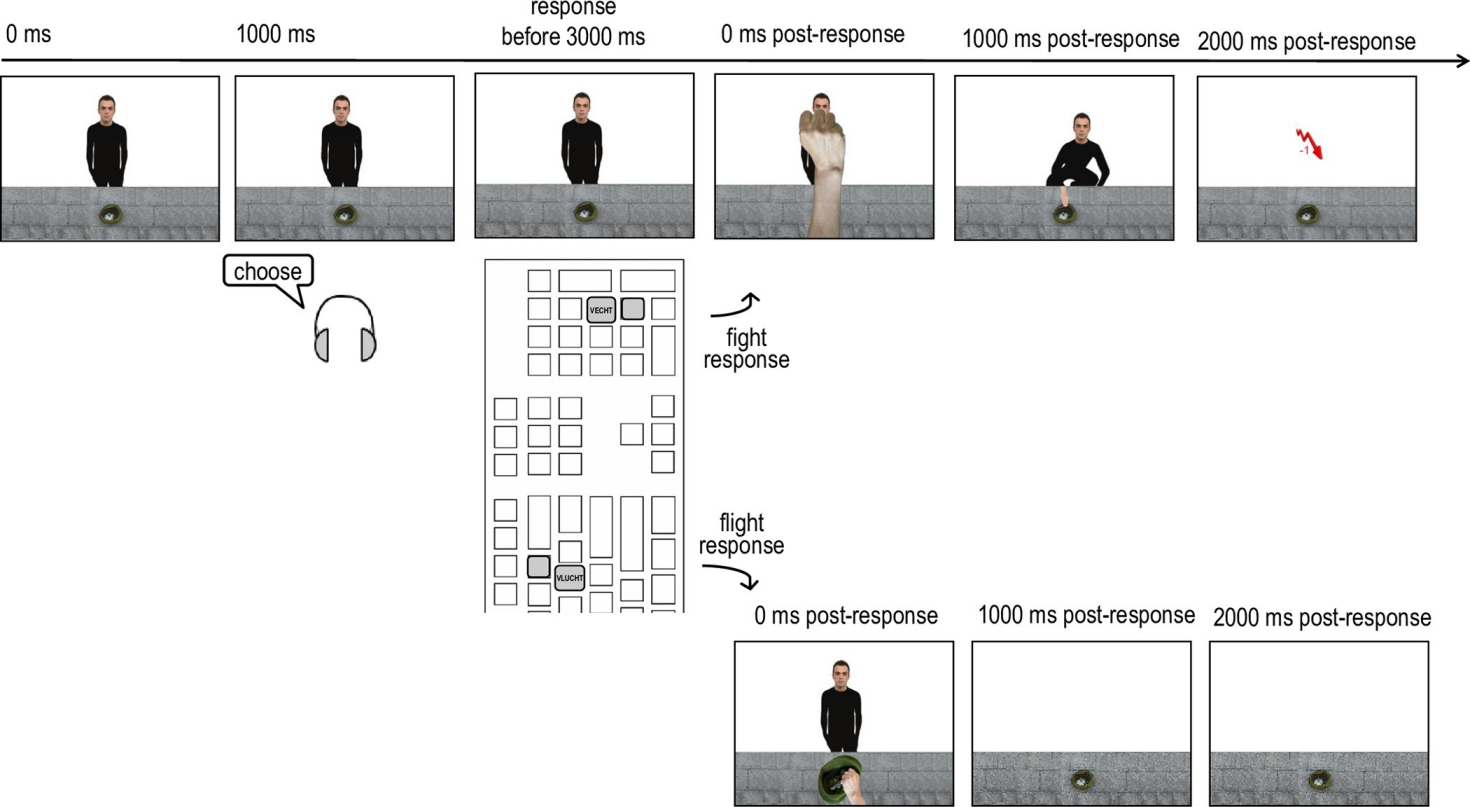
Order of events in a response trial of the test phase in the easy-flee condition with the right-fight-left-flee hand-R mapping. The instruction to choose was presented at 1000 ms post-stimulus onset and participants had to respond before 3000 ms post-stimulus onset. A fight response was followed by a fist punching the avatar, but the avatar nevertheless crouched and stole the money at 2000 ms post-stimulus onset. A flee response was followed by a hand grabbing the hat.

Participants were presented with the same four avatars as in the practice phases. They were again asked to keep their fingers on the keyboard without responding unless a cue was given. In this phase, participants no longer received fight and flee cues on any of the trials. Instead they received a choose cue (“kies” in Dutch) on half of the trials at 1000 ms after presentation of the avatar, after which they had to choose to either fight or flee. The sequence of events after each response was identical to that in the S:R-O practice phase (Fig 2), except that now real money was lost (1 euro) or won (2 euro). If participants fought against or fled from the giver during the response trials, they did not receive money from the giver on that trial. Participants could keep track of their score via a counter that appeared below the feedback (“+2” or “-1”) on each trial.

**Fig 3:**
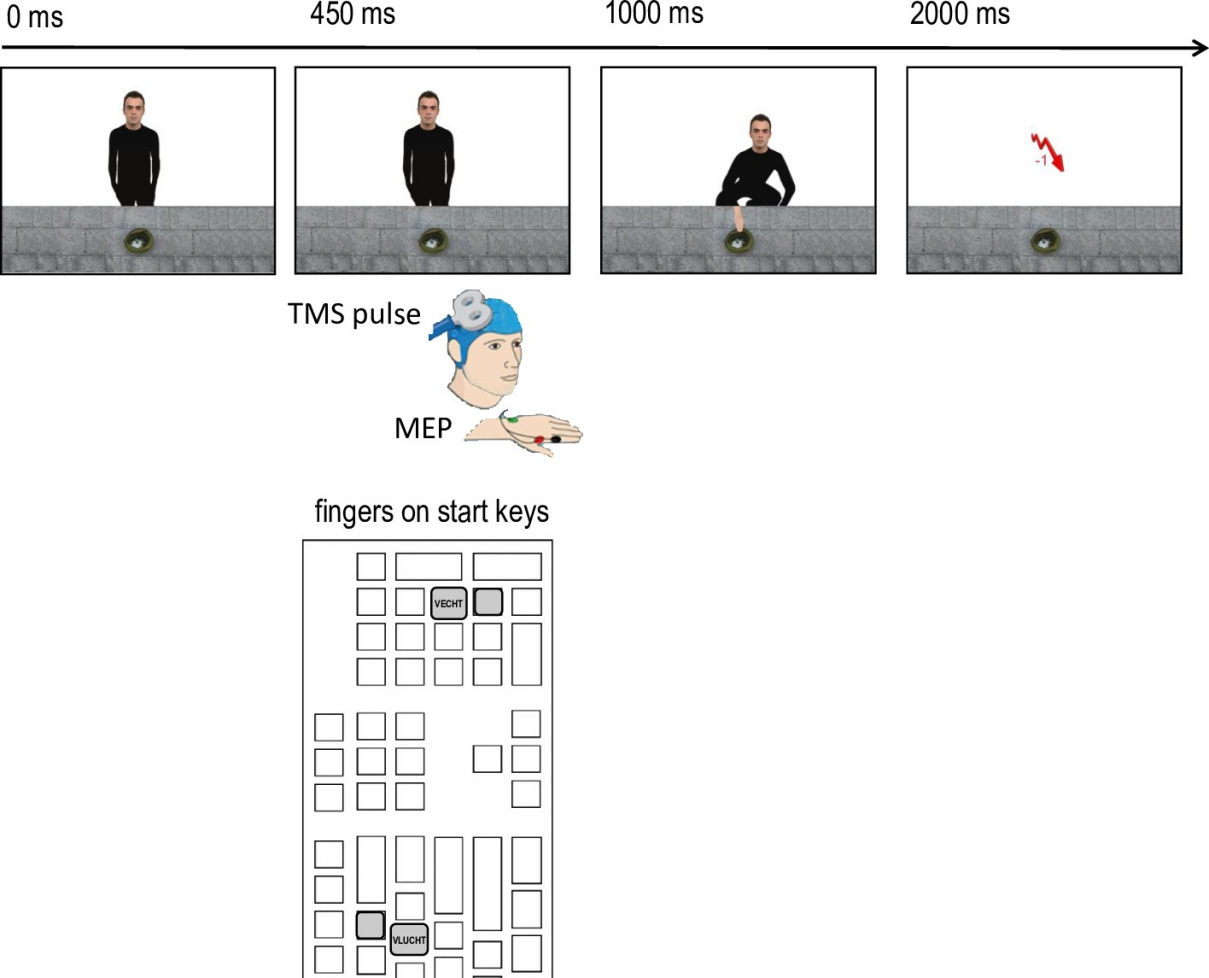
Order of events in an observation trial of the test phase in the easy-flee condition with the right-fight-left-flee hand-R mapping. After a TMS pulse at 450 ms, the avatar crouched and stole the money at 2000 ms post-stimulus onset.

Response trials were randomly intermixed with pure observation trials on which no cue was given and hence no response was required (Fig 3). Instead, they received a single TMS pulse to the motor cortex at 450 ms after presentation of the avatar. MEP amplitudes were measured online on the FDI muscles of the index fingers of both hands. Because participants were not allowed to respond on the observation trials, they received money from the giver and got stolen by each of the three thieves. The timing of events (crouching of giver and thieves and feedback) was the same as in the S:R-O phase.

Although previous research was able to detect that valenced stimuli led to a decrease in MEPs with a TMS pulse around 150 ms post-stimulus onset and an increase in MEPs around 300 ms [32], we opted for a slightly later timing of the TMS pulse for two reasons. First, the stimuli used in our study were more complex in the sense that participants had to first recognize the identity of the face of the avatar before being able to either appraise him as goal incompatible and easy/difficult to control (as per the stimulus-driven account) or to process the action option that had the highest expected utility in their presence (as per the goal-directed account). ERP research suggests that the brain does not respond to visual face identity until 230 after face onset [49]. Second, our study was set up to capture specific action tendencies to fight and flee, rather than a general increase or decrease of MEPs. Taken together, we estimated that the higher complexity of the stimuli as well as the higher level of specificity of the action tendencies would be reflected in a slightly later activation of these action tendencies. Piloting with the stimulus material of our study confirmed that a TMS pulse of 450 ms post-stimulus onset yielded the highest MEPs overall (i.e., independent of condition).

The test phase was composed of two blocks of trials. In each block, the coil was placed either to the left or the right hemisphere. The order of blocks (left first, right first) was counterbalanced across participants. During the observation trials of each block, TMS was applied and MEPs were registered from the index fingers of the contralateral hand. Each block comprised 120 trials: 60 response trials (15 giver, 15 difficult, 15 easy-fight, 15 easy-flee) intermixed with 60 observation trials (15 giver, 15 difficult, 15 easy-fight, 15 easy-flee). Participants were randomly assigned to one of two hand-R mappings. For participants who had the right-fight-left-flee mapping, MEPs were registered from the fight hand only in the coil-left block, and from the flee hand only in the coil-right block. For participants who had the right-flee-left-fight mapping, MEPs were registered from the flee hand only in the coil-left block, and from the fight hand only in the coil-right block.

#### Post-test phase

After the experiment, participants were presented with the pictures that had followed the fight and flight responses (i.e., the punching fist and the hat-grabbing hand) and they were asked to rate the valence of these responses on a visual analog scale, ranging from 1 (totally negative) to 100 (totally positive). In addition, they were presented with the faces of all avatars in random order and were asked to rate the valence, the physical strength, and the criminal look of each avatar using scales ranging from 1 (totally negative, totally weak, not at all criminal) to 100 (totally positive, totally strong, totally criminal). Finally, participants received the money they had won (30 euro) during the test phase before they were debriefed and thanked.

#### Measurements

In each response trial, we registered whether participants chose to overtly fight or flee. In each observation trial, we registered the TMS-induced MEPs. TMS pulses were delivered by a biphasic magnetic stimulator (Rapid2; Magstim). A 70 mm figure of eight coil was held tangentially to the skull with the handle pointing backwards and laterally at a 45° angle to the sagittal plane. The coil was positioned in correspondence with the optical scalp position defined as the coil position eliciting the largest and most reliable MEPs in the first dorsal interosseous (FDI) muscle. Participants wore a swimming cap on which the optimal location for stimulation was marked. A mechanical arm held the TMS coil and the experimenter continuously monitored the coil position. The stimulation intensity was determined based on the resting motor threshold (rMT) of the muscle, which is defined as the intensity that evokes a MEP larger than 50 μV in 50% of the cases in the FDI [31]. Stimulation intensity during the recording session was set to 110% of the rMT. Average intensity was 66.6% (range 44%-84%) of the maximal stimulator output for the right hemisphere and 66.8% (range 50%-82%) for the left hemisphere.

Electromyographical (EMG) activity was recorded with the ActiveTwo system (BioSemi). Sintered 11 × 17 mm active Ag–AgCl electrodes were placed over the FDI muscles of the right and left index fingers. These muscles contribute to abduct the index fingers away from the middle. The active electrodes were placed over the belly of the right/left FDI and the reference electrodes over the right/left ipsilateral proximal interphalangeal joints (belly-tendon montage). The ground electrode was placed on the back of the hand, near the wrist joint. The EMG signal was amplified (internal gain scaling), digitized at 2 kHz, high-pass filtered at 3 Hz, and stored on a PC for offline analysis.

## Results

We restricted our analyses to the measurements taken during the experimental trials for the three thieves in the three conditions: easy-fight, easy-flee, and difficult. The condition with the giver was not considered in the analyses. In fact, this condition was only inserted to allow participants to gain money and thus to keep their motivation high during the task. We first report the results for the manipulation checks from (a) the post-experimental questionnaires and (b) the frequency proportions of the fight and flee responses in the response trials. Next, we report the neurophysiological results: the MEP amplitudes for the fight and flee responses in the observation trials.

### Manipulation checks

#### Questionnaire data

Most participants (93%) correctly identified the three thieves at the end of the S-O practice phase. Only two participants made a mistake, which they corrected when the experimenter asked them to try again. This indicates that all participants eventually learned the S-O relations.

Analyses of the manipulation check data were carried out with 29 instead of 30 participants, because the manipulation check data of one participant could not be retraced. Repeated measures ANOVAs (with Greenhouse-Geisser correction for violation of sphericity) confirmed that our manipulation influenced the valence of the avatars, *F*(3, 40.65) = 71.99, *p* < 0.01, *η*^*2*^_*p*_ = .72. Bonferroni-corrected contrasts with a corrected α = .0125 indicated that the giver was rated as more positive (*M* = 80.79, *SD* = 19.44) than the three thieves (20.25, SD 17.62), F(1, 28) = 88.64, *p* < .001, *η*^*2*^_*p*_ = .76, but that the three thieves did not differ amongst each other (*M*_*easy-fight*_ = 22.59, *SD* = 17.16; *M*_*easy-flee*_ *=* 20.55, *SD* = 20.85; *M*_*difficult*_ = 17.62, *SD* = 20.37), all Fs < 4.20. The avatars also differed significantly with regard to physical strength, *F*(3, 69.96) = 4.32, *p* < .0125, *η*^*2*^_*p*_ = .13. This time, Bonferroni-corrected contrasts with a corrected α = .0125 indicated that the giver (*M* = 59.59, *SD* = 22.16) did not differ from the thieves (*M* = 55.75, *SD* = 15.31), *F*(1, 28) = 0.47, *p* = .50, *η*^*2*^_*p*_ = .02, but the thieves differed significantly amongst each other. The thief that was difficult to control was rated as physically stronger (*M =* 67.45, *SD* = 25.70) than the one that was easy to control by fighting (*M* = 43.14, *SD* = 26.79), *F*(1, 28) = 8.87, *p* < .0125, *η*^*2*^_*p*_ = .24, and than the one that was easy to control by fleeing (*M =* 56.66, *SD* = 26.66), although the latter difference did not reach significance, *F*(1, 28) = 3.40, *p* = .08, *η*^*2*^_*p*_ = .11. The latter two thieves showed no significant difference in physical strength, *F*(1, 28) = 4.30, *p* = .05, *η*^*2*^_*p*_ = .13. Avatars also differed with regard to criminal look, *F*(3, 46.13) = 53.63, *p* < .001, *η*^*2*^_*p*_ = .66. Contrasts revealed that the giver (*M* = 13.28, *SD* = 17.68) was rated as significantly less criminal than the thieves (*M* = 70.90 *SD* = 25.95), *F*(1, 28) = 70.41, *p* < .001, *η*^*2*^_*p*_ = .72, but no such difference was found among the three thieves (*M*_*easy-fight*_ = 67.24, *SD* = 26.98; *M*_*easy-flee*_ *= 72*.10, *SD* = 26.26; *M*_*difficult*_ *=* 73.35, *SD* = 30.57), all *Fs* < 3.07. Finally, the fight response (*M* = 26.76, *SD* = 27.53) was perceived as significantly more negative than the flee response (*M* = 60.38, *SD* = 23.00), *t*(28) = 4.34, *p* < .001, *d* = 0.81.

#### Choice frequencies

Visual inspection of the choice frequencies in Table 1 indicates that participants fought in nearly all trials of the easy-fight condition, and fled in nearly all trials of the easy-flee condition. In the difficult-control condition, there was more variation in the responses: Participants sometimes chose to fight or to flee, but they more often responded after the 2000 ms response deadline. To compare choice frequencies for fighting vs. fleeing across conditions, we calculated preference scores by substracting the number of flee responses from the number of fight responses for each participant, so that positive values reflected a preference for fighting and negative values a preference for fleeing. One-sample t-tests revealed a significant preference (as expressed by a preference score different from zero) for fighting in the easy-fight condition, *t*(29) = 132.46, *p* < .001, *d* = 24.18, a significant preference for fleeing in the easy-flee condition, *t*(29) = -225.93, *p* < .001, *d* = -41.27, and no significant preference for either response in the difficult condition, *t*(29) = 0.65, *p =* .52, *d* = .12. A one-way repeated measures ANOVA (with Greenhouse-Geisser correction for violation of sphericity) on these preference scores with within-subjects factor condition (easy-fight, easy-flee, difficult) yielded a significant effect, *F*(2, 29.75) = 504.69, *p* < .001, *η*^*2*^_*p*_ = .95. Planned comparisons revealed a significantly higher preference for fighting in the easy-fight condition (*M* = 29.33, *SD* = 1.21) than in the easy-flee condition (*M* = -29.63, *SD* = 0.72), *F*(1, 29) = 35602.77, *p <* .001, *η*^*2*^_*p*_ > .99. They also revealed that in the difficult condition (*M* = 1.43, *SD* = 12.16), the preference for fighting was significantly lower than in the easy-fight condition (*M* = 29.33, *SD* = 1.21), *F*(1, 29) = 145.55, *p <* .001, *η*^*2*^_*p*_ = .83, but significantly higher than in the easy-flee condition (*M* = -29.63, *SD* = 0.72), *F*(1, 29) = 197.02, *p <* .001, *η*^*2*^_*p*_ = .87.

**Table 1:** Frequencies (and percentages) of response choices and preference scores.

	Condition		
Response choices	Easy-fight	Easy-flee	Difficult
Fight	885 (98.55%)	4 (0.44%)	291 (32.37%)
Flee	5 (0.56%)	893 (99.22%)	248 (27.59%)
Preference score: fight—flee	880 (98.00%)	-889 (98.78%)	43 (4.78%)
Late	8 (0.89%)	3 (0.33%)	360 (40.05%)
Preference score: timely—late	882 (98,22%)	894 (99.33%)	179 (19.91%)

To compare choice frequencies for timely vs. late responses across conditions, we calculated preference scores by substracting the number of timely responses from the number of late responses for each participant, with positive values reflecting a preference for timely and negative values a preference for late responses. One-sample t-tests revealed a significant preference (as expressed by a preference score different from zero) for timely responses in both the easy-fight condition, *t*(29) = 114.66, *p <* .001, *d* = .21, and the easy-flee condition, *t*(29) = 267.46, *p <* .001, *d* = 48.85, but not in the difficult condition, *t*(29) = 1.31, *p =* .20, *d* = .24. A one-way repeated measures ANOVA (with Greenhouse-Geisser correction for violation of sphericity) on these preference scores with within-subjects factor condition (easy-fight, easy-flee, difficult) yielded a significant effect, *F*(2, 29.12) = 26.85, *p* < .001, *η*^*2*^_*p*_ = .48. Post-hoc comparisons showed smaller preferences for timely responses in the difficult condition (*M* = 5.97, *SD* = 24.88) than in the easy-fight condition (*M* = 29.40, *SD* = 1.40), *F*(1, 29) = 26.31, *p <* .001, *η*^*2*^_*p*_ = .48, and the easy-flee condition (*M* = 29.80, *SD* = 0.61), *F*(1, 29) = 27.47, *p <* .001, *η*^*2*^_*p*_ = .49.

### TMS/MEP

Neurophysiological data were processed offline using Matlab software. Epochs 500 ms before and after the TMS pulse were extracted from the continuous stream of data. Trials were rejected when the background EMG activity during the 500 ms interval preceding the TMS pulse was above 200 mV. Peak-to-peak MEP amplitudes (in mV) were calculated for the 20–50 ms window post-pulse. Trials with MEP amplitudes below 50 μV and outside of the range of +/- 2 SD from the average of each participant were discarded (14.44% of trials in the easy-fight condition, 14.41% of trials in the easy-flee condition, and 14.42% in the difficult condition), and the remaining amplitudes were normalized (*z*-scores) separately for FDI left and FDI right. Means were calculated for each response mapping (right-fight-left-flee, right-flee-left-fight), for each condition (easy-fight, easy-flee, difficult), and for each hand (right hand, left hand; see Table 2).

**Table 2:** Normalized mean MEPs (and SDs).

		Condition		
Hand-R mapping	Hand	Easy-fight	Easy-flee	Difficult
Right-fight-left-flee	Right hand	1.07 (0.16)	0.94 (0.18)	1.00 (0.13)
	Left hand	1.01 (0.13)	1.08 (0.17)	0.94 (0.12)
Right-flee-left-fight	Right hand	1.02 (0.12)	0.96 (0.15)	1.02 (0.15)
	Left hand	0.96 (0.09)	1.01 (0.09)	1.02 (0.10)

In a first step, we wanted to test whether the training of the hand-R mapping had been succesful on the TMS level. In order to do so, we conducted a 2 x 3 x 2 repeated measures ANOVA on the MEPs with between-subjects factor hand-R mapping (right-fight-left-flee, right-flee-left-fight) and within-subjects factors condition (easy-fight, easy-flee, difficult) and hand (right hand, left hand). This analysis did not reveal a significant interaction between these three factors, *F*(2, 27) = 0.88, p = .426, *η*^*2*^_*p*_ = .061, indicating that the training of the hand-R mapping had not been succesful on the TMS level (in dissociation with the overt behavioral level where this training had been succesful). This was not in line with our expectations.

A possible post-hoc explanation for this result is suggested by the well-established finding that approach behavior is preferentially executed with the right hand and avoidance behavior with the left hand [50]. This association between approach and a right-oriented bias and avoidance and a left-oriented bias has been explained by the lateralization of approach and avoidance tendencies in the brain: The tendency to approach/avoid is accompanied by increased left/right-hemispheric brain activity [50–52]. In our study, fighting involved approach whereas fleeing involved avoidance. Given the right/left-oriented bias for approach/avoidance behavior, it seems plausible to assume that the mapping of the fight response to the right hand and the flight response to the left hand was more natural than the mapping of the fight response to the left hand and the flee response to the right hand. It is possible that for participants who received the non-natural mapping instructions, the number of training trials to install the hand-R mapping was not sufficient to overcome the natural hand-R mapping, at least not at the early stage in which the TMS pulse was delivered.

Given that the training of the Hand-R mapping seems to have been ineffective regarding early action tendencies, we chose to conduct post-hoc exploratory analyses while coding the fight and flee hand to be in line with the natural mapping instead of the training mapping. This means that for participants in both hand-R mappings, the right hand was coded as the fight hand and the left hand as the flee hand. We also pooled the data across the two mappings.

The goal-directed and stimulus-driven accounts make two sets of contrasting predictions. The first set compares only the easy-flee and easy-fight conditions. Here, the goal-directed account predicts a condition x hand interaction, with MEPs in the fight hand higher than in the flee hand in the easy-fight condition, and MEPs in the flee hand higher than in the fight hand in the easy-flee condition. The stimulus-driven account, on the other hand, predicts a main effect of hand, with higher MEPs for the fight hand than for the flee hand, because both are easy conditions.

**Fig 4:**
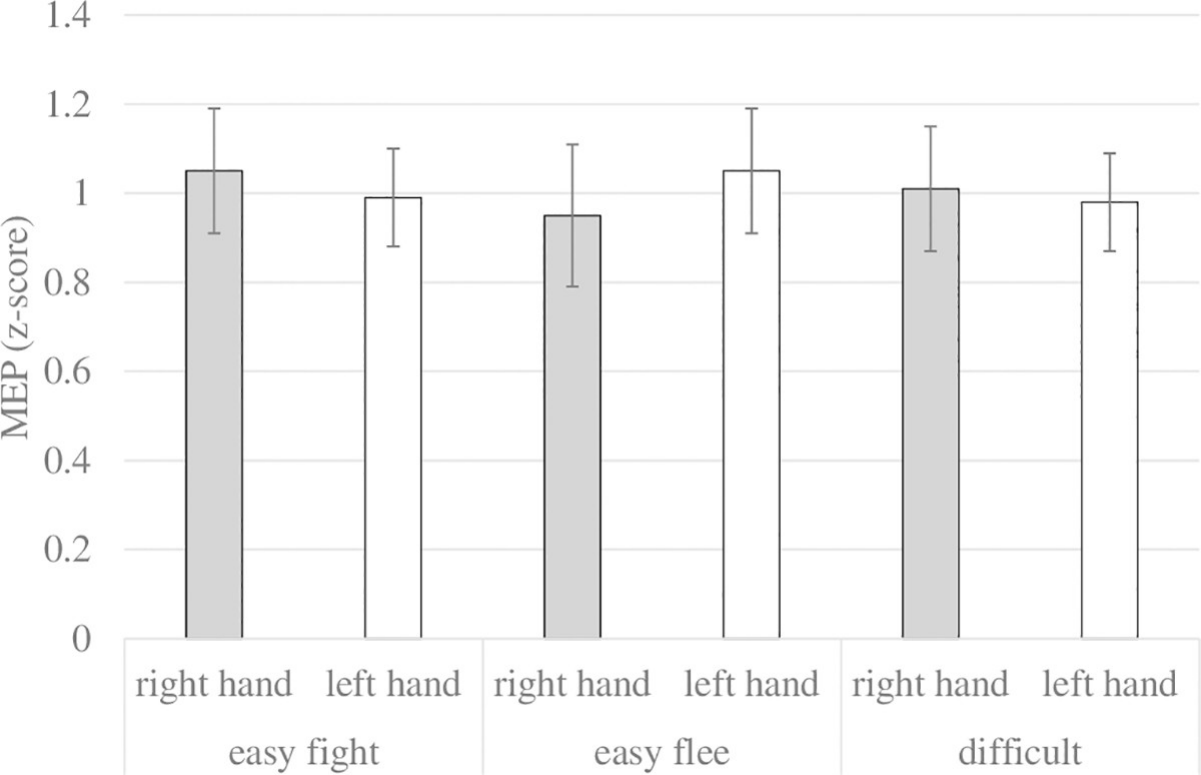
Normalized mean MEPs.

A test of the first set of predictions using a 2 x 2 repeated measures ANOVA with condition (easy-fight, easy-flee) and hand (fight hand, flee hand) as within-subject factors revealed a highly significant interaction effect, *F*(1, 29) = 13.10, *p* = .001, *η*^*2*^_*p*_ = .31, in line with the goal-directed account (see Fig 4). Planned comparisons were significant with means in the direction predicted by the goal-directed account: In the easy-fight condition, MEPs were higher for the fight hand (*M* = 1.05, *SD* = 0.14) than for the flee hand (*M* = 0.99, *SD* = 0.11), *F*(1, 29) = 4.87, *p* = .035, *η*^*2*^_*p*_ = .14, whereas in the easy-flee condition, MEPs were higher for the flee hand (*M* = 1.05, *SD* = 0.14) than for the fight hand (*M* = 0.95, *SD* = 0.16), *F*(1, 29) = 7.00, *p* = .013, *η*^*2*^_*p*_ = .19. The main effect for hand predicted by the stimulus-driven account could not be observed, nor was there a main effect of condition, all *F*s < 0.50.

The second set of predictions compares the difficult condition with each of the easy conditions (see also Fig 4). We first compared the difficult condition with the easy-fight condition. The goal-directed account predicts MEPs in the fight hand to be higher in the easy-fight than in the difficult condition (because the expected utility for fighting is higher in the former than the latter condition), but no difference between both conditions for the flee hand (because the expected utility of fleeing is zero in both). The stimulus-driven account also predicts MEPs in the fight hand to be higher in the easy-fight than in the difficult condition (because control is higher in the former, which should elicit a stronger tendency to fight), but MEPs in the flee hand to be higher in the difficult than in the easy-fight condition (because control is lower in the former, which should elicit a stronger tendency to flee). A 2 x 2 ANOVA with condition (easy-fight, difficult) and hand (fight hand, flee hand) yielded a significant main effect of hand with higher MEPs in the fight hand (*M* = 1.01, *SD* = 0.14) than in the flee hand (*M* = 0.98, *SD* = 0.11), *F*(1, 29) = 7.25, *p* = .012, *η*^*2*^_*p*_ = .20, but no significant condition x hand interaction, *F* < .31.

Next we compared the difficult condition with the easy-flee condition. Here, the goal-directed account predicts MEPs in the flee hand to be higher in the easy-flee than in the difficult condition (because the expected utility for fleeing is higher in the former than the latter), and no difference in MEPs between both conditions in the fight hand (because the expected utility of fighting is zero in both). The stimulus-driven account, on the other hand, predicts MEPs in the flee hand to be higher in the difficult than in the easy-flee condition (because control is lower in the former, which should elicit a stronger tendency to flee), and MEPs in the fight hand to be higher in the easy-flee than the difficult condition (because control is higher in the former, which should elicit a stronger tendency to fight). A 2 x 2 ANOVA with condition (easy-flee, difficult) and hand (fight hand, flee hand) yielded a significant interaction effect, *F*(1, 29) = 4.19, *p* = .043, *η*^*2*^_*p*_ = .134. Planned comparisons showed a trend effect in the flee hand with higher MEPs in the easy-flee (*M* = 1.05, *SD* = 0.14) than in the difficult condition (*M* = 0.98, *SD* = 0.11), *F*(1, 29) = 2.90, *p* = .099, *η*^*2*^_*p*_ = .09, in line with the goal-directed account. In the fight hand, MEPs were not significantly different between the easy-flee (*M* = 0.95, *SD* = 0.16) and difficult conditions (*M* = 1.01, *SD* = 0.14), *F* < 1.96, again in line with the goal-directed account. For the sake of completion, we also report a trend effect of hand, *F*(1, 29) = 3.37, *p* = .077, *η*^*2*^_*p*_ = .10, with higher MEPs in the flee hand (*M* = 1.01, *SD* = 0.13) than in the fight hand (*M* = 0.98, *SD* = 0.15), and no main effect of condition, *F* < .02.

We wish to add that if the data were coded according to the trained hand-R mapping, a similar pattern of results was obtained but only for participants in the right-fight-left-flee mapping. Data supporting the first set of predictions were somewhat less strong, but those supporting the second set of predictions were somewhat more outspoken (see S1 File for details).

## Discussion

In the current study, we pitted two mechanisms against each other that have been invoked to explain the early action tendencies to fight and flee. According to one stimulus-driven mechanism, high/low control over a goal-incompatible or negative stimulus activates the tendency to fight/flee. According to the goal-directed mechanism, the tendency to fight/flee is activated when fighting/fleeing has the highest expected utility. In typical cases, such as a competition in the animal world or between children on a playground, both mechanisms predict the same action tendency. This is because in these cases, fighting has the highest expected utility when control is high and fleeing when control is low. To pit the two mechanisms against each other, we created atypical cases in which both mechanisms predicted different action tendencies. Participants encountered three thieves (goal-incompatible stimuli) of which one could never be defeated (difficult to control and zero expected utility for fighting and fleeing), another could be defeated by fighting (easy to control and highest expected utility for fighting), and still another could be defeated by fleeing (easy to control and highest expected utility for fleeing). The said stimulus-driven process predicts a tendency to flee in the difficult condition and a tendency to fight in the two easy conditions. The goal-directed process predicts a tendency to be passive in the difficult condition, a tendency to fight in the easy-fight condition, and a tendency to flee in the easy-flee condition.

Participants’ overt choices were in line with the goal-directed hypothesis: They fought in nearly all trials of the easy-fight condition and fled in nearly all trials of the easy-flee condition. Moreover, they responded more often late than with fight or flight in the majority of the difficult trials, which can be understood as a sign of passivity and/or indecisiveness.

This result accords with, but goes also beyond two previous sets of findings. A first set shows that certain stimulus features (e.g., emotional quality or valence) are only processed when they are goal relevant (e.g., [53–54]). While this suggests that processing of these features is goal dependent, it does not yet show that the specific action tendencies activated are goal-directed in the sense that they depend on the values and expectancies of action outcomes. A second set of findings does show the role of goal-directed processes in emotional behavior (e.g., [55]). Solely relying on overt behavior, however, does not allow one to disambiguate between dual process models with (a) a default-interventionist architecture, in which an initial action tendency produced by a stimulus-driven process is overruled by a later action tendency produced by a goal-directed process, and (b) a parallel-competitive architecture, in which both processes operate in parallel and in which the goal-directed process wins the competition and determines the initial action tendency. Although both models predict that the eventual behavior will be caused by a goal-directed process, they each hold a different process to be responsible for the initial action tendency: a stimulus-driven process in the default-interventionist architecture, a goal-directed process in the parallel-competitive architecture.

To examine the initial action tendencies in the three conditions, we administered TMS shortly after presentation of the thieves and we measured EMG on the FDI muscles of two hands that were trained to fight and flee in a prior phase. A pre-analysis of our data suggested that the hand-R training mapping was not succesful at the TMS level. Leaning on the robust finding that approach/avoidance responses (including fighting/fleeing) are naturally executed with the right/left hand [50–52], we conjectured that the non-natural training installed in participants in the latter group was insufficient to override this natural mapping. Following the line of thought that all participants initially activated their right/left hand to fight/flee, we coded the right/left responses as fight/flee responses and conducted the analyses pooled across training mappings. As predicted by the goal-directed account, this yielded a strong condition (easy-fight, easy-flee) x hand (fight hand, flee hand) interaction effect, with significantly higher MEPs on the fight/flee hand in the easy-fight/easy-flee conditions. No evidence could be spurred for the main effect of hand predicted by the stimulus-driven account. In addition, a significant condition (easy-flee, difficult) x hand (fight hand, flee hand) interaction effect was found with higher MEPs on the flee hand in the easy-flee condition (in which fleeing had the highest expected utility) than the difficult condition (in which fleeing had zero expected utility) as predicted by the goal directed account, and not the reverse effect predicted by the stimulus-driven account.

Taken together, the behavioral results of our study yield support for the idea that people tend to fight or flee in order to obtain the outcome that is most beneficial to them, and that they tend to be passive when there is no beneficial action option available. These conclusions were corroborated by the neurophysiogical results of our study when the data of all participants were coded according to the presumed natural hand-R mapping. These findings suggest the operation of a goal-directed mechanism, instead of a stimulus-driven mechanism that links the general ease/difficulty to control an aversive stimulus to the tendency to fight/flee. The neurophysiological results, moreover, allow us to tentatively conclude that the goal-directed process already yields an action tendency as early as 450 ms post-stimulus onset. This is in line with the parallel-competitive scenario that places goal-directed mechanisms at the heart of the tendencies to fight and flee [2]. This presents a viable alternative to the widely endorsed default-interventionist scenario in which the goal-directed mechanism can only jump in at a later stage to regulate an initial stimulus-driven action tendency. Support for the early operation of goal-directed processes is also informative for other goal-directed models such as that of Mirabella [56], and Ridderinkhof [57] that present partial overlap with our own model.

We wish to point out that if the post-hoc explanation is correct that the participants in our study could not flexibly adjust the initially activated natural hand-R mapping in line with our instructions, this does not jeopardize our interpretation that the responses were goal-directed. This is because goal-directedness requires flexible adjustment of responses in accordance with current R-O mappings, which is different from the adjustment of hand movements in accordance with current hand-R mappings. Thus, our finding of a rigid hand-R relationship does not affect conclusions about the flexibility of the R-O relationships and hence of the goal-directedness of the responses. To drive this point home, compare with a case in which you are promised a million dollars if you write a particular sentence by using a different keyboard than the one you are used to (e.g., from querty to azerty). Chances are high that you want to write the sentence to obtain the money outcome, which suggests that the response of writing the sentence is goal-directed. It is likely, however, that you make mistakes in implementing this response in low-level motor movements.

It stands to reason, however, that even if participants were unable to adjust their hand-R mappings initially, as is reflected in the TMS/MEP data, they were still able to do so at a later time, as is indicated by the behavioral data. It seems then, that the initially rigid hand-R relationship could be flexibly corrected only at a later stage, while the fight/flee tendencies (R) themselves remained goal-directed throughout.

Evidence for the role of the goal-directed mechanism has implications for emotion theory. It has the potential to change the tenacious conviction that emotional behavior has an irrational flavor. Theorists who define emotional action tendencies in contrast with instrumental ones deny that the former can be instrumental (i.e., caused by goal-directed processes) on a priori grounds. They are likely to argue that the fight and flee tendencies that we measured do not qualify as emotional, precisely because the results suggest that they are instrumental. However, if emotional action tendencies are defined independent of a specific causal mechanism, the question as to which mechanism can cause these action tendencies is open to empirical research. For instance, if the criterion to call an action tendency more/less emotional is that it should be caused by a stimulus that is more/less goal relevant [2–3, 58–59], then both more/less emotional action tendencies can be caused by any mechanism. In the current study, the thieves are relevant and potentially incongruent with the goal to win or keep money. Thus, the fight and flee responses do qualify as emotional to some degree according to the criterion of goal relevance.

The mechanisms underlying emotional action tendencies also has implications for behavior change in clinical practice and society. If emotional action tendencies are caused by appraisal, unwanted emotional action tendencies should be changed by engaging in reappraisal. For instance, if aggressive tendencies are caused by the appraisal of a stimulus as goal incompatible but easy to control, unwanted aggressive tendencies can be toned down by teaching a person to reappraise the situation as less easy to control. If, on the other hand, emotional action tendencies are caused by a goal-directed process, unwanted emotional action tendencies should be changed by changing values and expectancies. If aggressive tendencies, for instance, arise because a person assesses that aggressive behavior has the highest expected utility for restoring a thwarted goal of high value (e.g., a status goal or a social norm), then unwanted aggressive tendencies can be toned down by teaching him/her that there are other, less costly action options for restoring that goal, such as lecturing the perpetrator.

A final contribution is methodological. The TMS method that we developed to answer our theoretical questions is innovative, both from the perspective of emotion research as from the perspective of motor TMS research. Traditional emotion research has measured emotional action tendencies via self-report [17], overt behavior [60], and stimulus-response compatibility tasks [30]. Our study contributes to recent attempts to extend the set of methods with motor TMS, which has the advantage that it can probe very early action tendencies. Previous TMS research inferred emotional action tendencies either from a general rise or decrease in action readiness (e.g., a decrease in MEP as an index of freezing; [32, 37]) or from specific muscle movements (e.g., an increase in MEP in FDI as an index of approach [44]). In our study, we did not infer but rather install the emotional meaning of muscle movements via a training phase in which these movements were instrumental for emotional actions (fight, flee). This method allowed us to measure tendencies to engage in these emotional actions in a more reliable way. There is a caveat, however. If we are right that the non-natural hand-R mapping trained in half of our participants was insufficient to override their natural hand-R mapping, three recommendations are in place. A first option is to only use mappings that are compatible with natural mappings. A second option is to establish arbitrary connections between responses and movements that do not have a strong pre-existing connection (e.g., use two fingers of the same hand). A third option might be to extend the number of training trials and/or install implementation intentions so as to override any natural connections.

Despite the strengths of the current study, a number of potential limitations deserve consideration. First, the study has been set up to challenge one particular stimulus-driven hypothesis (high/low control over a goal-incompatible stimulus leads to the tendency to fight/flee). Thus, our research does not speak to the validity of other stimulus-driven hypotheses.

Second, critics might argue that the reward structure of our study, which was conveyed to participants both via instructions and via experience during the S:R-O practice phase, could have led to the formation of temporary S-R links in which the representation of each thief is connected to the representation of a specific response. This fits with the notion of an implementation intention, which is understood as a temporary link between the representation of a stimulus and the representation of a response that the person wants to engage in [61]. For instance, a person who wants to avoid dental caries, can put up the implementation intention “Before bedtime (S), I will brush my teeth (R)”. Initially, researchers were convinced that once an implementation intention is put up, it acts autonomously from the overarching goal (e.g., avoiding dental caries) [62]. Recent research, however, shows that the overarching goal has to remain active in the background to avoid a breakdown of the implementation intention [63]. Applied to our study, if participants did put up temporary S-R links, it is likely that these links were not isolated from the overarching goal (e.g., to maximize profit), and hence that they cannot be considered as pure S-R links but rather as S-R links embedded in an overarching goal-directed process.

A third potential limitation concerns the timing of the TMS pulse. As explained in the method section, the TMS pulse was administered at 450 ms post-stimulus onset because we estimated that the higher complexity of the stimuli as well as the higher level of specificity of the action tendencies would result in a slightly later activation of these action tendencies than what has been found in certain previous studies [32]. We admit, however, that the current study does not completely rule out a scenario in which a stimulus-driven process occurred still before that time and was subsequently overruled by a goal-directed process, in line with a default-interventionist architecture. Future studies could be conducted with slightly earlier TMS timings and perhaps more sensitive measures of corticospinal excitability to further examine this issue.

A fourth issue concerns the timing of the response cues in the response trials at 1000 ms after the presentation of the avatar. At the time that the avatar is presented, participants do not know whether the trial will be a response trial or an observation trial, so they are likely to prepare a response prior to the response cue. Shifting the response cue to a time prior to the presentation of the avatar would not have been an option, however, because if participants would know beforehand that a certain trial is an observation trial, they should also know that responding has zero expected utility, and hence a goal-directed process should predict passivity.

Summing up, the present paper presents a novel method based on motor TMS that has potential in disambiguating between stimulus-driven and goal-directed accounts of early action tendencies. Using this method, we obtained preliminary support for the goal-directed account rather than for one specific stimulus-driven account of the early tendencies to fight and flee. The evidence is not conclusive, however. Future research should examine this issue further, using a wider range of TMS pulse times.

## Supporting information

S1 File Analyses when data were coded according to the trained hand-R mapping. (DOCX)

S1 Fig Normalized mean MEPs for the right-fight-left-flee hand-R mapping. (TIF)
