# Supplementary material for: The role of stimulus-driven versus goal-directed processes in fight and flight tendencies measured with motor evoked potentials induced by Transcranial Magnetic Stimulation
Source: PLoS One. 2019 May 20;14(5):e0217266. doi: 10.1371/journal.pone.0217266 (PMC6527228; doi:10.1371/journal.pone.0217266)
Supplement: S1 File — (DOCX) [file pone.0217266.s001.docx]

This section describes the analyses performed on the data if these data are coded according to the trained hand-R mappings. For participants in the right-fight-left-flee mapping, the right hand is coded as the fight hand and the left hand as the flee hand, whereas for participants in the right-flee-left-fight hand, the right hand is coded as the flee hand and the left hand as the fight hand.

In a first step, we conducted a 2 x 3 x 2 repeated measures ANOVA on the MEPs with between-subjects factor hand-R mapping (right-fight-left-flee, right-flee-left-fight) and within-subjects factors condition (easy-fight, easy-flee, difficult) and hand (fight hand, flee hand). This analysis revealed a significant interaction between these three factors, *F*(2, 56) = 5.20, *p* = .008, *η^2^_p_* = .16. No other interactions or main effects were significant, all other *F*s < 1.64.

In order to explore the three-way interaction, we analyzed the data for the two hand-R mappings separately. The goal-directed and stimulus-driven accounts make two sets of contrasting predictions. The first set compares only the easy-flee and easy-fight conditions. Here, the goal-directed account predicts a condition x hand interaction, with MEPs in the fight hand higher in the easy-fight than the easy-flee condition, and MEPs in the flee hand higher in the easy-flee than the easy-fight condition. The stimulus-driven account, on the other hand, predicts a main effect of hand, with higher MEPs for the fight hand than for the flee hand, because both are easy conditions.

In the right-fight-left-flee mapping, a 2 x 2 ANOVA with condition (easy-flee, easy-fight) and hand (fight hand, flee hand) yielded a significant interaction effect, *F*(1, 14) = 9.42, *p* = .008, *η^2^_p_* = .40, with means in the predicted direction: In the easy-fight condition, MEPs were higher for the fight hand (*M* = 1.07, *SD* = 0.16) than for the flee hand (*M* = 1.01, *SD* = 0.13) whereas in the easy-flee condition, MEPs were higher for the flee hand (*M* = 1.08, *SD* = 0.17) than for the fight hand (*M* = 0.94, *SD* = 0.18). Planned comparisons showed that only the latter contrast reached significance, F_easy-fight_ (1, 14) = 1.52, *p* = .237, *η^2^_p_* = .10; F_easy-flee_ (1, 14) = 7.72, *p* = .015, *η^2^_p_* = .36. Nevertheless, the significant interaction effect does suggest a differential expression of the MEPs for fighting and fleeing depending on the expected utilities of these responses, in line with the goal-directed account. The main effect for hand predicted by the stimulus-driven account could not be observed, nor was there a main effect of condition, *F*s < 1.44.

The 2 x 2 ANOVA performed for the right-flee-left-fight mapping, on the other hand, did not yield any significant effects. Although a trend towards a hand x condition interaction could be spurred, *F*(1, 14) = 3.95 , *p* = .067, *η^2^_p_* = 0.22, the means involved in this interaction were opposite to the prediction of the goal-directed account: In the easy-flee condition, MEPs were higher for the fight hand (*M* = 1.01, *SD* = 0.09) than for the flee hand (*M* = 0.96, *SD* = 0.15); in the easy-fight condition, MEPs were higher for the flee hand (*M* = 1.02, *SD* = 0.12) than for the fight hand (*M* = 0.96, *SD* = 0.09). Neither of these contrasts reached significance, although the first contrast came close, *F*_easy-fight_(1, 14) = 4.05, *p* = .064, *η^2^_p_* = .22; *F_easy-flee_*(1, 14) = 0.90, *p* = .360, *η^2^_p_* = .06.

The second set of predictions compares the difficult condition with each of the easy conditions. We first compared the difficult condition with the easy-fight condition. The goal-directed account predicts MEPs in the fight hand to be higher in the easy-fight than in the difficult condition (because the expected utility for fighting is higher in the former than the latter condition), but no difference between both conditions for the flee hand (because the expected utility of fleeing is zero in both). The stimulus-driven account also predicts MEPs in the fight hand to be higher in the easy-fight than in the difficult condition (because control is higher in the former, which should elicit a stronger tendency to fight), but MEPs in the flee hand to be higher in the difficult than in the easy-fight condition (because control is lower in the former, which should elicit a stronger tendency to flee). In the right-fight-left-flee mapping, a 2 x 2 ANOVA with condition (easy-fight, difficult) and hand (fight hand, flee hand) yielded a significant main effect of hand with higher MEPs in the fight hand (*M* = 1.03, *SD* = 0.15) than in the flee hand (*M* = 0.97, *SD* = 0.12). A significant condition x hand interaction could not be observed, *F* < .01. In the right-flee-left-fight mapping, the same ANOVA did not yield any significant effects, all *F*s < 2.27.

Next we compared the difficult condition with the easy-flee condition. Here, the goal-directed account predicts MEPs in the flee hand to be higher in the easy-flee than in the difficult condition (because the expected utility for fleeing is higher in the former than the latter), and no difference in MEPs between both conditions in the fight hand (because the expected utility of fighting is zero in both). The stimulus-driven account, on the other hand, predicts MEPs in the flee hand to be higher in the difficult than in the easy-flee condition (because control is lower in the former, which should elicit a stronger tendency to flee), and MEPs in the fight hand to be higher in the easy-flee than the difficult condition (because control is higher in the former, which should elicit a stronger tendency to fight). In the right-fight-left-flee mapping, a 2 x 2 ANOVA with condition (easy-flee, difficult) and hand (fight hand, flee hand) yielded a significant interaction effect, *F*(1, 14) = 5.383, *p* = .036, *η^2^_p_* = .278. Planned comparisons showed that MEPs in the flee hand were significantly higher in the easy-flee (*M* = 1.08, *SD* = 0.17) than in the difficult condition (*M* = 0.94, *SD* = 0.12), *F*(1, 14) = 4.71, *p* = .048, *η^2^_p_* = .25, in line with the goal-directed account. MEPs in the fight hand were not significantly different between the easy-flee (*M* = .94, *SD* = 0.18) and difficult conditions (*M* = 1.00 , *SD* = 0.13), *F* < 1.19, again in line with the goal-directed account. In the right-flee-left-fight mapping, the same ANOVA did not yield any significant effects, all *F*s < 1.03.

Taken together, the goal-directed account received preliminary support, but only for participants who fought with the right hand and fled with the left hand. The MEPs of participants who received the opposite hand-R mapping did not differ significantly across conditions. A trend interaction effect between condition (easy-fight, easy-flee) and hand (fight hand, flee hand) for these participants even turned out to be opposite to the predictions of the goal-directed account.
