# Supplementary figures and images for: The role of stimulus-driven versus goal-directed processes in fight and flight tendencies measured with motor evoked potentials induced by Transcranial Magnetic Stimulation
Source: PLoS One. 2019 May 20;14(5):e0217266. doi: 10.1371/journal.pone.0217266 (PMC6527228; doi:10.1371/journal.pone.0217266)

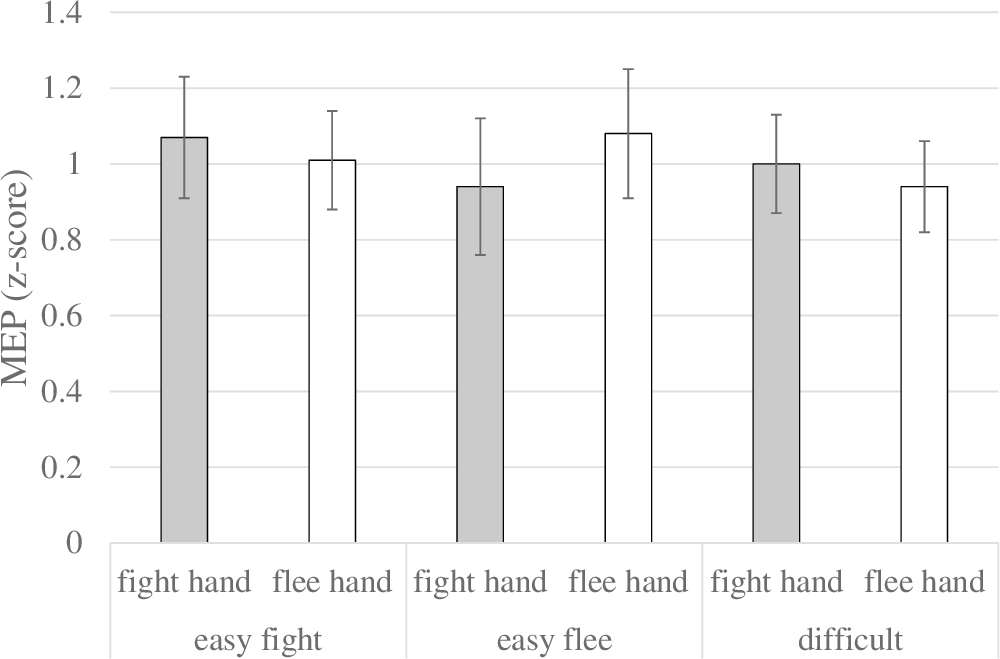

Supplement: S1 Fig — (TIF) [file pone.0217266.s002.tif]
